# Auricular acupressure for insomnia in hemodialysis patients: study protocol for a randomized controlled trial

**DOI:** 10.1186/s13063-018-2546-2

**Published:** 2018-03-07

**Authors:** Yuchi Wu, Lihong Yang, Lingli Li, Xiuqing Wu, Zhicong Zhong, Zhiren He, Hongyan Ma, Lixin Wang, Zhaoyu Lu, Cun Cai, Daixin Zhao, Xiangxin Meng, Airong Qi, Aicheng Yang, Guobin Su, Xinfeng Guo, Xusheng Liu, Chuan Zou, Qizhan Lin

**Affiliations:** 1 Hemodialysis Department, Guangdong Provincial Hospital of Chinese Medicine, Guangzhou, People’s Republic of China; 2 Second Clinical Medical College, Guangzhou University of Chinese Medicine, Guangzhou, People’s Republic of China; 3 Evidence-based Medicine and Clinical Research Service Group, Guangdong Provincial Academy of Chinese Medical Sciences, Guangzhou, People’s Republic of China; 4 Hemodialysis Department, Guangzhou Charity Hospital, Guangzhou, People’s Republic of China; 5 Hemodialysis Department, Guangzhou HEMC (Higher Education Mega Center) Hospital, Guangzhou, People’s Republic of China; 6 Hemodialysis Department, Guangzhou Hospital of Traditional Chinese Medicine, Guangzhou, People’s Republic of China; 7 Hemodialysis Department, Shenzhen Hospital of Traditional Chinese Medicine, Shenzhen, People’s Republic of China; 8 Hemodialysis Department, Wuyi Hospital of Traditional Chinese Medicine, Jiangmen, People’s Republic of China

**Keywords:** Insomnia, Hemodialysis, Auricular acupressure, Randomized controlled trial

## Abstract

**Background:**

Patients on maintenance hemodialysis (MHD) frequently complain of insomnia. Poor sleep quality impairs their quality of life and adversely affects long-term outcome. Previously we applied auricular acupressure therapy (AAT) for MHD patients with insomnia and yielded favorable results. AAT probably improves sleep quality by stimulating the vagus nerve and inhibiting sympathetic overactivity. However, the efficacy of AAT for insomnia in this population is still lacking. The proposed randomized controlled trial (RCT) will evaluate the efficacy and safety of AAT for improvement of sleep quality in MHD patients with insomnia.

**Methods/design:**

The proposed study is a multi-center, double-blind (participants and assessors), parallel-group RCT. A total of 112 participants with insomnia will be recruited from six hemodialysis centers in Guangzhou, China, and randomly allocated in a 1:1 ratio to receive auricular acupressure on either active points (AA group) or control points (points irrelevant to insomnia management, SAA group). The treatment will last for 8 weeks prior to a follow-up period of 12 weeks. Evaluation by blinded assessors at baseline, at 8 weeks (end of treatment) as well as at 4-week, 8-week and 12-week follow-ups (after intervention) will include Pittsburgh Sleep Quality Index (PSQI) scores and average weekly dose of hypnotics. The primary endpoint is clinical response rate (percentage of participants who reach a reduction of PSQI global score ≥ 3 in each group) at 8 weeks from baseline. Secondary endpoints include the changes in PSQI scores over time from baseline, as well as the changes in weekly dose of hypnotics.

**Discussion:**

This paper describes the rationale and design of a double-blind RCT that aims to determine the efficacy and safety of AAT for insomnia of hemodialysis patients. If successful, this project will provide evidence of the efficacy and safety of AAT for insomnia of hemodialysis patients.

**Trial registration:**

ClinicalTrials.gov, Identifier: NCT03015766. Registered on 22 December 2016.

**Electronic supplementary material:**

The online version of this article (10.1186/s13063-018-2546-2) contains supplementary material, which is available to authorized users.

## Background

Sleep disorders, such as insomnia, restless leg syndrome, sleep apnea and excessive daytime sleepiness, are common in patients with end-stage renal disease (ESRD), especially in those receiving dialysis [1]. Insomnia is one of the most common conditions, with a prevalence rate as high as 49–84.5% depending on the specific population [2–5]. Poor sleep quality is known to have adverse effects on patients’ health-related quality of life [6], mental health [7], social adaptability [8] and even long-term survival [9].

ESRD patients with insomnia share common risk factors identified in the general population, such as female sex, advanced age, lack of physical activity and depressed mood [10]. In addition, multiple factors, including chronic kidney disease (CKD) and renal replacement therapy, also play roles in the development of insomnia. CKD comorbidity (e.g., diabetes) and CKD-related complications (e.g., uremia, anemia and hyperparathyroidism) are all recognized factors [5, 11, 12]. Somatic symptoms, such as pruritus, bone pain and arthralgia, are also related to impaired sleep quality in dialysis patients [13].

Poor sleep quality prompts physicians to prescribe hypnotics (usually benzodiazepines) for hemodialysis (HD) patients, which may be associated with adverse effects, such as memory impairment, drug resistance, dependence and addiction [14]. Current guideline for chronic insomnia suggests that sleep medications should be properly used based on awareness of both effectiveness and harms [15]. Despite concerns regarding safety and dependency of long-term usage of hypnotic drugs expressed by both physicians and patients, a considerable proportion (benzodiazepines: 42.6% and hypnotics: 20.0%, in single-center cross-sectional study [16]) of HD patients continue to take nightly sleep medications for prolonged periods while at the same time complaining of unsatisfactory sleep quality. Therefore, both caregivers and patients are confronted with the conflicting requirements of improving patients’ sleep quality and reducing their dependence on hypnotics.

Non-pharmacological therapies with supportive evidence of a hypnotic effect are desirable. Auricular acupressure therapy (AAT), a therapeutic approach that treats various disorders by stimulating specific points on the ear, is a popular form of complementary medicine in China. Kung’s study in Taiwan among women with postmenopausal insomnia indicated that AAT led to increased cardiac parasympathetic activity and reduced cardiac sympathetic activity with improvement of sleep quality [17]. In another investigation in women with postpartum insomnia [18], patients receiving AAT for 2 weeks showed decreases in Pittsburgh Sleep Quality Index (PSQI) total scores from 8.7 (pre test) to 5.57 (post test, 36% reduction). Scores on the subscales of PSQI also showed significant improvement of sleep quality. Autonomic nervous system regulation by the auriculovagal afferent pathway [19] has been proposed to explain how AAT improves sleep quality. The auricular branch of the vagus nerve links the acupressure stimulation and vagal regulation. AAT might trigger vagal regulation and correct the overactivity of sympathetic nerves which plays a crucial role in chronic hemodialysis patients with insomnia [20]. Therefore, auricular acupressure might be effective in improving sleep quality for hemodialysis patients.

Our previous study showed improvements in sleep quality and reduced consumption of sleep medications (6.98 ± 4.44 pills per week post AAT compared with 4.23 ± 2.66 pills per week pre AAT, *P* < 0.01) after a 4-week course of AAT in addition to basic care in hemodialysis patients with severe insomnia [21]. In a pilot randomized controlled trial (RCT), we found that AAT on specific points with an appropriate rationale yielded a higher response rate in PSQI score improvement than that on sham points (62.5% vs. 32.3%, *χ*^2^ = 5.77, *P* = 0.02) [22]. However, these previous studies had some limitations. Data collected from a single center may lack the scientific rigor or external validity required to support widespread application of AAT. In addition, sample size was not calculated to guarantee sufficient statistical power. Owing to design limitations, the efficacy and safety of AAT in hemodialysis populations with insomnia have yet to be confirmed. Therefore, we aim to perform a full-scale RCT to test the hypothesis that AAT is an effective and safe means of improving sleep quality and reducing dependence on hypnotics in hemodialysis patients.

## Methods/design

### Study design

The study uses a two-arm, randomized, double-blind, sham-controlled design. The trial will be performed in six hemodialysis centers in Guangdong Province, People’s Republic of China, all of which are tertiary hospitals of Chinese Medicine: Guangdong Provincial Hospital of Chinese Medicine, Guangzhou Charity Hospital, Guangzhou HEMC (Higher Education Mega Center) Hospital, Guangzhou Hospital of Traditional Chinese Medicine, Wuyi Hospital of Traditional Chinese Medicine and Shenzhen Hospital of Traditional Chinese Medicine. This trial will include an 8-week treatment period and a 3-month follow-up period (visits at 4-week intervals). Eligible subjects will be randomized into one of the two arms in a ratio of 1:1 to receive AAT on either active points or sham points. The trial has been registered at ClinicalTrials.gov (NCT03015766).

### Participants

**Fig. 1:**
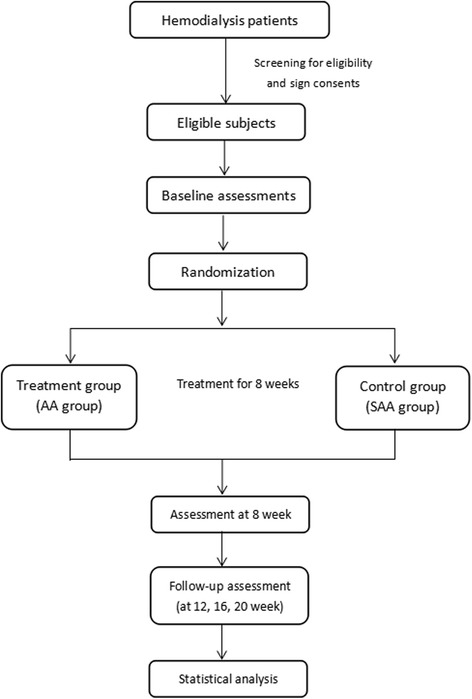
Trial flow chart

We will include patients receiving regular hemodialysis treatment for more than 3 months and less than 10 years. These patients are more likely to go through the whole trial which will continue for 5 months. Incident hemodialysis patients (less than 3 months) and those who have been on dialysis for more than 10 years usually have a high mortality rate [23, 24]. Patients will have initial assessment of sleep quality and physical examination to meet the diagnosis of chronic insomnia. Potentially eligible participants are being identified through screening of patient medical records at each dialysis center. Screening of patients in each center will be performed by one assigned personnel according to the inclusion and exclusion criteria. A flow chart of the participant recruitment is shown in Fig. 1.

### Participant timeline

**Fig. 2:**
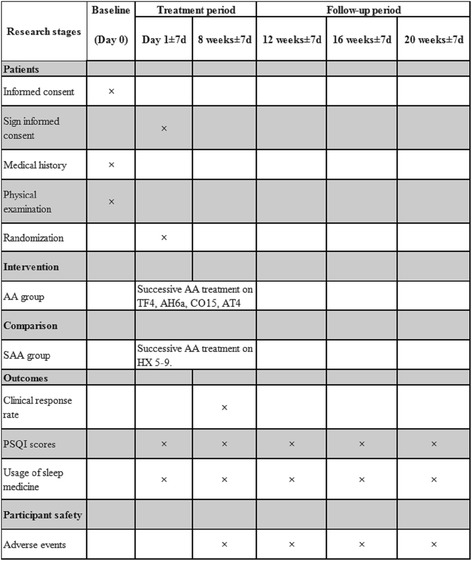
Standard Protocol Items: Recommendations for Interventional Trials (SPIRIT) Figure. Timing of visits and data collection. Abbreviations: AA auricular acupressure, SAA sham auricular acupressure, PSQI Pittsburgh Sleep Quality Index

The time course for participant recruitment, intervention, assessment and follow-up is shown in the Standard Protocol Items: Recommendations for Interventional Trials (SPIRIT) Figure (Fig. 2).

### Inclusion criteria

Subjects fulfilling all of the following criteria will be included:

1. Aged 18–75 years (either sex).
2. On regular hemodialysis (two or three sessions every week, 4 h each session, total weekly dialysis period ≥ 10 h)
3. Insomnia diagnosed according to *The Diagnostic and Statistical Manual of Mental Disorders, Fifth Edition* (DSM-5) [25]
4. Baseline global PSQI score > 7
5. Informed consent provided

### Exclusion criteria

Patients with any of the following conditions will be excluded:

1. Presence of comorbidities including cancer, congestive heart failure, connective tissue disease and hematological diseases
2. Inadequately dialyzed, indicated by urea clearance index (KT/V) < 1.20
3. Presence of severe physical symptoms, such as bone pain, itchy skin, sleep apnea and restless legs, which are obviously causative of insomnia; and exhaustion caused by severe anemia (hemoglobin < 60 g/L) or malnutrition (serum albumin < 30 g/L)

### Randomization and allocation concealment

Subjects will be randomly allocated into one of the two treatment arms in a 1:1 ratio: (1) treatment group (AA group) and (2) control group (SAA group). Central randomized allocation will be performed. Patients will be stratified based on six sites and randomly assigned to one of the two groups. The Chinese Clinical Trial Registry will generate the random number sequence using computer software, and will deposit the allocation sequence in ResMan^®^ Clinical Trial Management Public Platform, a web-based database. When a new participant is enrolled in the study, the research coordinator of each site will apply for the allocation information through this online system and assign participants to interventions accordingly. It is not possible to speculate on the allocation status of the participant before obtaining this information. The other personnel, including research nurses, assessors and clinical physicians, are not authorized to apply for random numbers. All processes will be recorded and saved appropriately.

### Blinding

Both the participants and the assessors in this trial will be blinded to the allocation. AAT will be provided by trained nurses and manipulated by patients. The nurses and patients will be told that we are comparing two different treatment protocols (TPs), in which all auricular points involved are named as numbers with specific defined locations. We will have two nurses in each site assigned to perform the intervention. One will be responsible for providing TP A and the other for TP B. The assignment of nurses is done by drawing lots. The nurses will receive researcher training before the trial. They are required to perform auricular acupressure following the standard procedure, no matter what TP they are practicing. They are also required not to discuss the difference between the two TPs with patients. To avoid differences due to the Hawthorne and Rosenthal effect [26, 27], we will script the nurses to limit their interaction with the participants. Any additional questions will be directed to the research coordinator of each center. All results will be reported to another investigator responsible for assessing sleep quality in a clinical interview. The investigator will provide questionnaires to participants at the scheduled time for assessment. Clinical physicians of the Hemodialysis Department are allowed to prescribe estazolam (14 tablets each time) when patients require. At the end of the week, the patients should return the tablets left and obtain a new prescription if they need. All personnel will be properly assigned before the start of the trial.

### Interventions

**Fig. 3:**
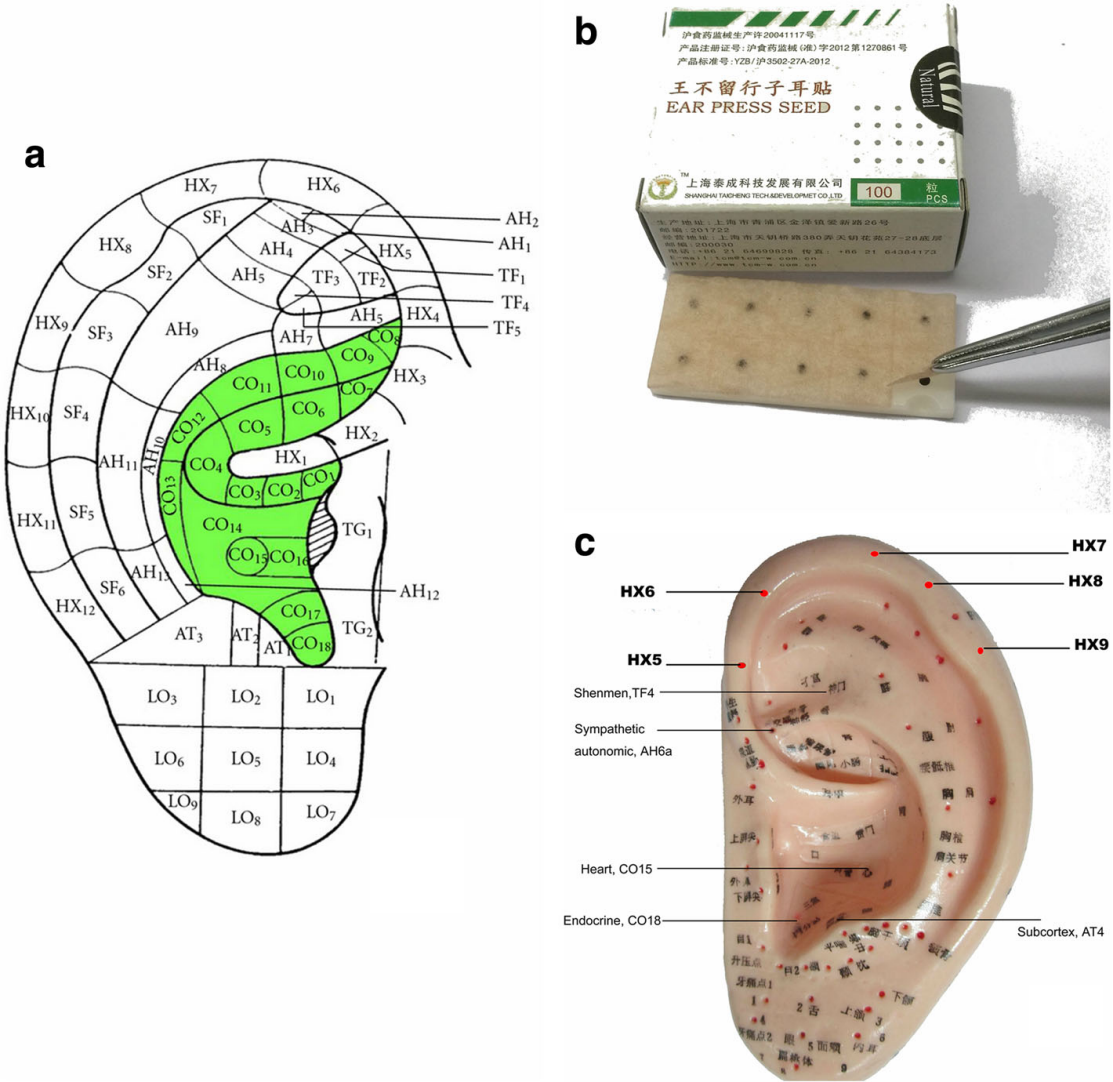
A demonstration of interventions in the trial. **a** A map of auricular acupoints commonly used. **b** Ear-press seed (Wang Bu Liu Xing) applied in this trial. **c** Acupoints of both groups in the trial

AAT is based on the theory that specific points on the auricle correspond to major organs or systems of the body and that the function of the targeted organ or system can be modulated by manipulating corresponding auricular acupoints. AAT applies stimulation through pressure on specific acupoints using embedded beads, usually *Semen vaccariae* seeds (*Wang Bu Liu Xing*) or stainless steel beads. This approach is non-invasive, and recipients can be trained to manipulate the beads themselves to exert stimulation at a required frequency. A map of commonly used auricular acupoints is shown in Fig. 3a.

In this trial, two research nurses for each center will be assigned to administer AAT to participants. They will gain approval from licensed acupuncturists through training and competence assessment before the start of the trial. They will provide AAT for patients during dialysis sessions for eight consecutive weeks. A standard procedure of AAT has been established as follows:

1. Identify the ear points according to the TP directed by the research coordinator.
2. Sterilize the involved area of the ear with 75% isopropyl alcohol
3. Attach an adhesive plaster (size: 1.0 cm × 1.0 cm) with one bead (*Semen vaccariae*, globes approximately 2.0 mm in diameter; surface: smooth; colour: black; Taicheng Technology & Development Co., Ltd., Shanghai, China) embedded in each acupoint (Fig. 3b)
4. Provide instructions to press the beads with appropriate strength (approximately 0.3–0.4 kg) and rate (one to two beats per second) for 1 min at each point [28]. This should be done three times during the day and once in the evening
5. The plasters will be replaced with fresh ones every 2–3 days (usually on the dialysis day) depending on the dialysis schedule. If the plasters or beads become detached, participants should come to the hospital to receive fresh plasters. To improve their adherence to interventions, we plan to attach the auricular acupressure beads when the patients come for their scheduled dialysis and replace the beads every 2–3 days (on the dialysis day as well). Activities during each visit in the treatment period will be documented in the Case Report Form.

### Treatment group

Participants in the treatment group (AA group) will receive AAT on five active acupoints:

- Acup.1. *Shen Men* (Spiritual Gate, TF4)
- Acup.2. *Jiao Gan* (Sympathetic autonomic, AH6a)
- Acup.3. *Xin* (Heart, CO15)
- Acup.4. *Pi Zhi Xia* (Subcortex, AT4)
- Acup.5. *Nei Fen Mi* (Endocrine, CO18)

The rationale for selecting these points has been explicitly described previously [21, 22]. Briefly, these points were selected based on Traditional Chinese Medicine (TCM) meridian theory, results of an academic literature review, and on-site clinical practice experience [29]. These points help patients sleep by regulating the autonomic nervous system. These points are anatomically connected to the auricular branch of the vagus nerve. Stimulation on these points may increase parasympathetic activities and reduce sympathetic activities [30, 31], which play an important role in sleep [32].

### Control group

Participants in the control group (SAA group) will receive AAT on five helix points (HX 5–9), which are clearly remote from the inner ear area. These points lack evidence for insomnia management. In our pilot study, these points had minor effects on sleep quality, which could not be differentiated from the placebo effect.

The acupoints in both groups are illustrated in Fig. 3c. The locations of these points in both groups are listed in Table 1, following the National Standards of the Nomenclature and Location of Auricular Acupoints published in China [33].

**Table 1:** Locations of auricular acupoints used in the trial

Auricular acupoints	Locations
AA group	
TF4	At the apex of the triangular fossa, in the bifurcating point between superior and inferior crura of the antihelix
AH6a	At the end of the inferior antihelix crus
CO15	Around the central depression of the cavum conchae
AT4	At the medial side of the antitragus
CO18	In the intertragic notch, at the medial inferior part of the cavum conchae
SAA group	
HX 5	An area on the helix, between the two feet of the antihelix
HX 6	An area on the helix, between the upper foot of the antihelix and the apex conchae auris
HX 7	An area on the helix, between the apex conchae auris and the helix tubercle
HX 8	An area on the helix, and at the helix tubercle
HX 9	An area on the helix, and below the helix tubercle

*AA* auricular acupressure, *AAT* auricular acupressure therapy

### Sample size estimation

In a pilot RCT conducted previously, the clinical response rates of AAT and sham AAT were 62.5% and 32.3%, respectively [22]. With these data, we used the following formula to calculate the estimated sample size [34].

$$ \mathrm{N}\kern0.5em =\kern0.5em \frac{{\left({u}_{\alpha }+{u}_{\beta}\right)}^2\left(1+\raisebox{1ex}{$1$}\!\left/ \!\raisebox{-1ex}{$\mathrm{k}$}\right.\right)p\left(1-p\right)}{{\left({p}_e-{p}_c\right)}^2},\kern0.5em p\kern0.5em =\kern0.5em \frac{p_e+{kp}_c}{1+k} $$

In this formula, *k* (the ratio of allocation) = 1, *u*_a_ and *u*_*β*_ are 1.6449 and 1.2816, respectively, when 90% statistical power (*β* = 0.1) is required and a significance level of 5% (*α* = 0.05) is allowed. Here, *p*_e_ represents the incidence rate of the treatment group, while *p*_c_ represents that of the control group. Therefore, we will require at least 56 subjects in each group taking into account an allowed dropout rate of 15%. The results have been confirmed using PASS software 11.0 (NCSS, LLC, Kaysville, UT, USA) by tests for two independent proportions.

### Outcome measurement

Sleep quality will be measured by PSQI score [35]. PSQI contains seven domains with scores from 0 to 3, yielding a total score ranging from 0 to 21, where a higher score indicates poorer sleep quality. The reliability and validity of its Chinese version have been confirmed [36, 37]. PSQI will be assessed at baseline, immediately after treatment (i.e., 8 weeks from baseline) and at 4, 8 and 12 weeks after treatment.

#### Primary outcome measurement

The primary outcome will be the difference of the clinical response rates at 8 weeks from baseline between two groups. According to the academic literature review, response is defined as a reduction of PSQI global score ≥ 3 [38]. The clinical response rate will be the number of participants who achieve a reduction of PSQI global score exceeding 3 points divided by the total number of participants in each group and multiplied by 100%.

#### Secondary outcome measurement

Secondary outcomes will be changes in PSQI scores (including global score and scores of each of the seven domains) at the end of treatment and at each follow-up visit compared with baseline.

As the trial will not exclude participants on hypnotics or plan a washout period due to ethics considerations, participants may maintain their dose of hypnotics or require hypnotic agents during the study. In this case, clinical physicians will prescribe estazolam (14 tablets each prescription, 1 mg each tablet) and instruct them to adjust the dose of hypnotics according to their sleep quality. The participants should return the unconsumed tablets at the end of the week or require another prescription as needed. The weekly dose of hypnotic agents will be reported and also serve as a secondary outcome.

#### Patient safety

All participants will undergo blood biochemical tests before randomization and at the end of follow-up. According to Blood Purification Standard Operating Procedure in China [39], maintenance hemodialysis (MHD) patients will undergo investigations including serum creatinine (SCr), blood urea nitrogen (BUN), total carbon dioxide (TCO_2_), potassium, calcium, phosphate, parathyroid hormone (PTH), albumin, hemoglobin and KT/V every 3 months. These parameters are all essential for monitoring the complications and adequacy of dialysis for MHD patients. We will utilize these data to assess their safety during the trial. Adverse events throughout the treatment and follow-up periods, regardless of relevance to the interventions, will be documented, reported to the Ethics Committee and dealt with using appropriate treatment.

### Withdrawal criteria

Participants will be withdrawn from the study in the following situations:

1. When a participant asks to withdraw from the study, at any time, for any reason
2. When severe adverse events or reactions occur

The data of these participants will be collected and included in further analysis.

### Data collection and management

Data of demographic characteristics and baseline assessment will be collected by screeners when the patients are recruited. Clinical outcome measurement will be performed by assigned outcome assessors after the treatment is completed and during the follow-up period. Assessors of each center will undergo a training course to obtain the capability of using PSQI. Data regarding medicine prescription and any adverse events reported by patients will be collected by clinical physicians of the Hemodialysis Department.

A research coordinator of each center will conduct quality control of data collection and be responsible for data entry. The data manager will be responsible for initial data cleaning, identifying, coding and conversion into the proper format for data analysis. Data analysis will be performed by a biostatistician from the Evidence-based Medicine and Clinical Research Service Group.

### Statistical analysis

Statistical analysis will be performed using SPSS Statistics Software 22.0 (IBM Corp., Armonk, NY, USA). For the results of the study, means ± standard deviations will be calculated for normally distributed quantitative variables and median (interquartile range) for non-normally distributed variables. In addition, the 95% confidence interval around the mean will be presented. For categorical variables, the number and percentage of patients within each category will be presented.

Statistical analyses of primary and secondary outcomes will be conducted as follows. First, all analyses will be based on the intention-to-treat (ITT) principle. All patients enrolled in the study will be included in the analyses, regardless of whether they complete the treatment or adhere to the protocol. Data distribution is expected to be normal, and skewed distribution data will be transformed prior to analysis. Then, the outcome of the clinical response will be analyzed by the c*hi*-squared test or Fisher’s exact test. For the secondary outcomes, repeated-measures analyses of variance (rANOVA) will be conducted to compare the changes in PSQI scores and the weekly dose of hypnotics at 8, 12, 16 and 20 weeks from baseline. Missing values will be addressed using the method of last observation carried forward (LOCF). To avoid potential confounding factors, additional sensitivity analyses will be performed following these statistical analyses.

## Discussion

Impaired sleep quality of patients on dialysis adversely impacts their quality of life and long-term survival. Although AAT has been practiced as a complementary treatment for insomnia, there is still an important gap with regard to sufficient evidence. The results of this trial are expected to provide convincing evidence regarding whether AAT is effective for MHD patients with insomnia.

This study has been designed carefully following the Consolidated Standards of Reporting Trials (CONSORT) Statement of RCTs and reported according to the Standard Protocol Items: Recommendations for Interventional Trials (SPIRIT) Statement (Additional file 1). One of the major issues in the trial design is the setting of the control group. Studies on the efficacy of acupuncture have been controversial based mainly on the following two points: first, the psychological effect (placebo effect, from both the investigators and the patients) could make a considerable contribution to the results. Second, the therapeutic effect may not depend on specific points. Our previous pilot study showed that points on the helix, which are irrelevant to the physiology of sleep, are appropriate for the sham intervention. In addition, we specified the points in both groups as numbers and letters. We also scripted the interactions between practitioners/assessors and subjects. These approaches help to blind both the participants and the practitioners, reducing their psychological interference to the minimum. Therefore, it will be easier to interpret the contributions of specific acupoints to the outcome.

Another concern is whether hypnotics should be washed out and prohibited during the trial. Indeed, this approach is good for trial design but impractical for carrying out the trial, because it increases the suffering of patients and reduces their compliance. As the trial is intended to assess whether AAT can help improve sleep quality and reduce drug use, rather than whether AAT is better than hypnotics, we decided to allow the use of hypnotics under conditions of strict surveillance.

For outcome measurement, PSQI is a well-validated, patient-reported questionnaire. But for now there is not a defined minimal clinically important difference (MICD) to reflect the changes in a clinical intervention that are meaningful for the patients. In some case, statistically significant difference has little clinical significance. Therefore, we adopted the response rate for the primary endpoint from a clinical trial published by Prof. Buysse, the author of the PSQI. He performed a comprehensive data review to set the criteria for response: participant with a change in PSQI of more than 3 points after treatment was defined as a response case [38]. In addition, the global PSQI score and scores of seven domains will be also analyzed as secondary outcomes in this trial.

The design of the trial minimizes the risk of bias by the following methods: (1) It is designed as a multi-center, double-blinded RCT. A central randomization method is applied to ensure completely random allocation and concealment. Both the patients and the assessors are blinded to the groups, (2) The sample size was estimated based on a previous pilot study [22], (3) The screening of participants is performed strictly to represent the population studied, (4) The manipulation of AAT is standardized and the practice of AAT is performed strictly under the guidance of authoritative clinical guidelines [40, 41]. A senior acupuncturist will be invited to train the research nurses from six sites and assess their competence, which will ensure the homogeneity of treatment and (5) The selected scale for outcome measurement is of good validity and reliability [42]. The investigators involved in the study will undergo a training course and have their role assigned clearly before the start of the trial.

There are several limitations of this study. First, sleep quality is not assessed by objective measurement such as polysomnography or actigraphy. There are some difficulties for us to perform these tests. Most hemodialysis patients refused to go through polysomnography in our pilot studies when they were required to stay a whole night in hospital. Actigraphy seems easily accepted but this equipment is unavailable in all centers. Due to limited financial support, we have to make the best of a well-acknowledged scale to assess the outcome. In a future study, actigraphy should be included. Second, anxiety and depression are commonly accompanied with sleep disorders but are not assessed in this study. Recent study has suggested that they have positive correlations with PSQI score [43]. A more comprehensive assessment of the effect of auricular acupressure will include sleep quality, anxiety and depression, and quality of life in future study.

This protocol offers a standardized process to guide subsequent clinical research. The results of this trial will generate concrete evidence regarding the efficacy of AAT in insomnia management of dialysis patients.

### Trial status

Ongoing recruitment

## Additional files

Additional file 1: SPIRIT Checklist. SPIRIT 2013 Checklist: recommended items to address in a clinical trial protocol and related documents. (DOC 97 kb)

Additional file 2: Complementary information for the trial. (DOC 35 kb)

Additional file 3: WHO Trial Registration Data Set. (PDF 79 kb)

Additional file 4: Informed consent materials. (DOC 30 kb)

## Abbreviations

AA: Auricular acupressure
AAT: Auricular acupressure therapy
BUN: Blood urea nitrogen
CKD: Chronic kidney disease
CONSORT: Consolidated Standards of Reporting Trials
DSM: Diagnostic and Statistical Manual of Mental Disorders
ESRD: End-stage renal disease
HD: Hemodialysis
ITT: Intention-to-treat
LOCF: Last observation carried forward
MHD: Maintenance hemodialysis
PSQI: Pittsburgh Sleep Quality Index
PTH: Parathyroid hormone
RCT: Randomized controlled trial
SAA: Sham auricular acupressure
SCr: Serum creatinine
TCM: Traditional Chinese Medicine
TCO_2_: Total carbon dioxide
TP: Treatment protocol
