# Supplementary material for: Auricular acupressure for insomnia in hemodialysis patients: study protocol for a randomized controlled trial
Source: Trials. 2018 Mar 7;19:171. doi: 10.1186/s13063-018-2546-2 (PMC5842538; doi:10.1186/s13063-018-2546-2)
Supplement: Supplementary file 2 — Complementary information for the trial. (DOC 35 kb) [file 13063_2018_2546_MOESM2_ESM.doc]

***Complementary information for the trial***

1. **[item 3]Date and version identifier**

Date: 18 Sep, 2017

Version identifier: Version 1/20170918

1. **[item 5b]Name and contact information for the trial sponsor**

Sponsor: The State Administration of Traditional Chinese Medicine, P. R. China and Guangdong Provincial Hospital of Chinese Medicine (YN2015MS25).

Contact information:

Tel: +86-010-59957777 Add: No.1 Gongti West Rd., Dongcheng District, Beijng, China.

Tel:+86-020-81887233 Add: No.111, Dade Rd., Guangzhou, China.

1. **[item 5d]Composition, roles, and responsibilities of the coordinating centre, steering committee, endpoint adjudication committee, data management team, and other individuals or groups overseeing the trial, if applicable**

The Scientific Research Management Department of Guangdong Provincial Hospital of Chinese Medicine will be in charge of trial auditing and inspection for the study. It will have contracted Clinical Research Organization to oversee the trial. A program manager and his team will visit each site to examine trial procedures, ensure data quality, and monitor compliance with the protocol every 3 months. A data safety monitoring board, consisting of seven independent members, will be established to assess safety events, primary outcomes, and data quality.

Professor Qizhan Lin is responsible for making major decisions for protocol development. This trial will be performed in six centers. Each center has a research co-ordinator. He/she will be responsible for screening participants, applying for random number and allocation results, and inputting data into the ResMan online system. He/she will contact the principle investigator (Qizhan Lin) or the research co-ordinator (Yuchi Wu) of the project for any problem during the trial.

The Case Report Forms are designed by Yuchi Wu. All data will be initially recorded in the Case Report Forms and inputted into the ResMan online system (http://www.medresman.org) by the assigned personnel of each center. The database will be managed by Yuchi Wu and Lihong Yang who is from Evidence-based Medicine and Clinical Research Service Group.

1. **[item 10]Inclusion and exclusion criteria for participants.**

Inclusion and exclusion criteria for participants are reported in the main text. To protect patients’ privacy, we will generate a name code using four capital letters, which are captured according to the Chinese phonetic alphabet of the patients’ names by scheduled rules. For example, Zhou Yu is coded as “ZHYU”; and Wu Mei-qi is coded as WMQI. The coding rules are stated in the Case Report Forms and will be one of the training contents for researchers.

1. **[item 11c]strategies for maintaining adherence to intervention protocols**

• Renew of the auricular acupressure beads will be performed when the patients come for their scheduled dialysis.

• A timetable for participants’ scheduled day for hemodialysis and auricular acupressure is designed.

• Research nurses will remind the participants of the next day for treatment when they provide the intervention.

• If anyone fails to come for any reason, physicians of Hemodialysis Department will phone him/her or his/her family to follow up.

• The participants are required to return the left tablets (sleep medications) at the end of each week, which will be counted and recorded by their physicians.

• Medical expense for investigations involved in this trial will be covered.

1. **[item 15]Strategies for achieving adequate participant enrollment to reach target sample size**

• posting recruitment advertisements in the waiting area of each hemodialysis center;

• communicating more information on sleep quality with hemodialysis patients through diverse ways like patient education activities and mobile messages;

• sharing best practice with our Research Nurses,

• evaluating the sleep quality of hemodialysis patients every three months.

1. **[item 17b]Under which circumstances, is unblinding permissible? And the procedure for revealing a participant’s allocated intervention during the trial.**

Unblinding should occur only in exceptional circumstances when knowledge of the actual treatment is absolutely essential for further management of the patient. However, the intervention studied, i.e. auricular acupressure, is a supplement to essential treatment. The likelihood that unblinding in this trial is necessary would be minimal, although we could assumed that some extreme complications including psychosis, sudden death and suicide might happen.

Investigators should firstly discuss with the Medical Advisor if unblinding is necessary. If so, the investigator should apply through ResMan® system for emergency unblinding. He will be informed of with the actual intervention information of the involved participant. Code breaks should be documented in the Case Report Form as they occur.

1. **[item 18b]Plans to promote participant retention and complete follow-up.**

**Strategies to optimize participant retention**

• Provide educational material on insomnia management to patients.

• Collect information available in their medical profiles, only ask questions that are necessary

• Select questionnaires with lesser response time without compromising the result.

• Make appointments for outcome assessment on the day they come for dialysis.

• Complete questionnaires using mobile phone or IPAD.

**Strategies to minimize loss to follow-up**

• Train committed research staff

• Keep the duration between screening and enrollment short.

• Maintain a log of all events

• Use an online tracking system to schedule visits

• Immediately follow up on missing or incomplete information.

• Identify the loss to follow-up patients, and make an effort to locate them.

• Assign research personnel to coordinate the trial at each site.

1. **[item 19]Plans for data entry, coding, security, and storage to promote data quality.**

• Data entry and coding

Case Report Forms completed by each branch center should be sent to the host research center(The second Affiliated Hospital of Guangzhou University of Chinese Medicine) in 15 working days. Two person are assigned as data entry clerks to examine the completeness and accuracy of the data and establish database by double-entry. After double-entry, check the coherence, correct any error according to the original data. All data were input to online system. Blind review of data will be performed at the end of research.

• Data security and storage

After data entry completes, the database is lock and not allowed for modification. All files by all materials are collected and recorded to CD-ROM for long term preservation.

1. **[item 21a]Data monitoring committee (DMC)**

DMC as well as interim analyses are not planned for the following reasons:

• The treatment period of the trial is relatively short.

• Previous studies have showed potential benefits for the interventions and minor risk for participants.

• The intervention studied is complementary for insomnia management.

**7. [item 23]Frequency and procedures for auditing trial conduct, if any, and whether the process will be independent from investigators and the sponsor**

• Frequency：every two months

• Procedures: (1) create an audit team, which involves staff from GCP office of Guangdong Provincial Hospital of Chinese Medicine; (2) development of an audit plan through discussions with program faculty; (3) completion of the audit and (4) audit follow-up.

• The process will be independent from investigators and the sponsor.

1. **[item 25]Plans for communicating important protocol modifications (eg, changes to eligibility criteria, outcomes, analyses) to relevant parties**

• Ethics Committee will review and follow up the research protocol every year.

• When there are any important protocol modifications, we will report to onsite Ethics Committee for approval and updated it on ClinicalTrials.gov.

• After it is approved, we will inform the research co-ordinator of each center by both email and phone. The modified protocol with a new version identifier will be delivered to all centers. If it is necessary, the principle investigator (Qizhan Lin) will have someone go to each center for researcher training.

1. **[item 26a]Who will obtain informed consent or assent from potential trial participants or authorized surrogates, and how?**

The project has been approved locally by Ethical Committee of Guangdong Provincial Hospital of Chinese Medicine (Document No: B2016-137-01). Research coordinator of each site will obtain informed consent or assent from potential trial participants. We will display our research plan through videos and leaflets and invite potential trial participants through mobile messages, telephone or face-to-face interaction. Informed consent will be obtained from participants after they read and understand the purpose, benefits and risks that may arise in the research. They will take sufficient time to make their own decisions. Whether the decision is agree to participate or not will not have any influence on their due treatment in hemodialysis center. They will sign the informed consent in a quiet and pressure-free situation.

1. **[item 27]Confidentiality of research data**

• Although data collection of patient information should be accurate, the analysis

of data does not require the identification of subjects.

• Electronic data should be password protected and hard/paper data should be kept in a locked cabinet. Research data will only be used by authorized personnel of the research team, which is assigned by the principle investigator.

• Computer-to-computer transmission is preferred when transmitting data is needed. If it has to be via e-mail, the database should be submitted to encryption.

**11. [item 29]Access to data**

The principle investigator (Qizhan Lin) and the assigned statistician will have access to the final trial dataset. Contractual agreements among research sites as well as the ResMan system user authentication will limit unallowed access to the data.

**12. [item 30]Compensation to those who suffer harm from trial participation**

For those who suffer harm from the trial, compensations according to the guide of Good Clinical Practice(GCP) Act. Medical treatment for the adverse event will be covered and additional financial compensation will be paid with consensus.
