# Supplementary material for: Auricular acupressure for insomnia in hemodialysis patients: study protocol for a randomized controlled trial
Source: Trials. 2018 Mar 7;19:171. doi: 10.1186/s13063-018-2546-2 (PMC5842538; doi:10.1186/s13063-018-2546-2)
Supplement: Supplementary file 3 — WHO Trial Registration Data Set. (PDF 79 kb) [file 13063_2018_2546_MOESM3_ESM.pdf]

This file includes answers to all items of WHO Trial Registration Data Set.

ClinicalTrials.gov Protocol Registration and Results System (PRS) Receipt
Release Date: May 23, 2017

ClinicalTrials.gov ID: NCT03015766

Study Identification

Unique Protocol ID: YN2015MS25

Brief Title: Auricular Acupressure for Hemodialysis Patients With Insomnia (AAHDIN)

Official Title: Auricular Acupressure for Hemodialysis Patients With Insomnia: Study Protocol for a Multi-centre Double-blind, Randomized Controlled Trial

Secondary IDs:

Study Status

Record Verification: May 2017

Overall Status: Recruiting

Study Start: May 1, 2017 [Actual]

Primary Completion: October 1, 2018 [Anticipated]

Study Completion: June 1, 2019 [Anticipated]

Sponsor/Collaborators

Sponsor: Guangdong Provincial Hospital of Traditional Chinese Medicine

Responsible Party: Principal Investigator
Investigator: Qizhan Lin [qlin]
Official Title: Professor
Affiliation: Guangdong Provincial Hospital of Traditional Chinese Medicine

Collaborators:

Oversight

U.S. FDA-regulated Drug: No

U.S. FDA-regulated Device: No

U.S. FDA IND/IDE: No

Human Subjects Review: Board Status: Approved
Approval Number: B2016-137-01
Board Name: Institutional Ethics Committee
Board Affiliation: Guangdong Provincial Hospital of Traditional Chinese Medicine
Phone: +86-20-81887233
Email: szyllwyh@163.com
Address:

No.111 Dade Rd. Guangzhou, 510120，P.R.China

Data Monitoring: No

Plan to Share IPD: Yes
IPD including the demographic information, PSQI scores and drug use at baseline and 8,12,16,20 week from baseline of all participants will be shared. The data will be availbale at ResMan® Clinical Trial Management Public Platform, with the link http://www.medresman.org/uc/index.aspx

FDA Regulated Intervention: No

Study Description

Brief Summary: Auricular acupressure therapy (AAT) has been applied in MHD patients with insomnia in recent years and yielded favorable results. However, the effect and safety of AAT for insomnia in MHD population still lacks high quality evidence. A randomized controlled clinical trial is planned to evaluate the effect and safety of AAT in MHD patients with insomnia.

Detailed Description: Insomnia, a worldwide health problem, is much more frequently complained in maintenance hemodialysis (MHD) patients and impairs their quality of life and long term outcome. Hypnotic sedative agents are often reluctantly prescribed with doses mounting up. Patients are concerned about drug dependence and drug-related adverse effects. As a non-drug therapy, auricular acupressure therapy (AAT) is attractive to both patients and practitioners and is widely used to treat many conditions in China. The investigators had been applying AAT for MHD patients with insomnia in recent years and yielded favorable results. However, the effect and safety of AAT for insomnia in MHD population still lacks high quality evidence. Therefore, the investigators aimed to perform a randomized controlled clinical trial in MHD patients with insomnia to evaluate the effect and safety of AAT.

Conditions

Conditions: Insomnia Chronic

Keywords: insomnia
hemodialysis
auricular acupressure
randomized controlled trial

Study Design

Study Type: Interventional

Primary Purpose: Treatment

Study Phase: N/A

Interventional Study Model: Parallel Assignment

Number of Arms: 2

Masking: Participant, Care Provider, Outcomes Assessor

Allocation: Randomized

Enrollment: 112 [Anticipated]

Arms and Interventions

Arms	Assigned Interventions
Experimental: Auricular acupressure therapy<br>Participants in the treatment group will received AAT on five active acupoints including Acup.1. Shen Men (Spiritual Gate, TF4), Acup.2. Jiao Gan (Sympathetic autonomic, AH6a), Acup.3. Xin (Heart, CO15), Acup.4. Pi Zhi Xia (Subcortex, AT4), Acup.5. Nei Fen Mi (Endocrine, CO18)	auricular acupressure therapy<br>Auricular acupressure, is a therapeutic method in which specific acupoints on the ear are stimulated to treat various disorders of the body. This practice is based on the theory that there are specific points on the auricle which correspond to major organs or systems of the body; and therapeutic effect on the corresponding target organ or system can be exerted by manipulating auricular acupoints. Auricular acupressure applies stimulation through pressure on specific acupoints by the imbedded beads, usually Semen Vaccaria (Wang Bu Liu Xing) or stainless steel beads. This therapeutic method is non-invasive and can be self-manipulated by the recipients at times required.<br>Other Names:<br>• auricular acupressure<br>• ear acupressure
Sham Comparator: sham auricular acupressure therapy<br>Participants in the control group (SAA group) will receive auricular acupressure on five Helix points (HX 5–9), which were clearly remote from the inner ear area. These points have no evidence for insomnia management.	sham auricular acupressure therapy<br>The intervention is the same as that in the experimental group only when the points are five Helix points (HX 5-9). These points are clearly remote from the inner ear area and have no evidence for insomnia treatment.<br>Other Names:<br>• sham AAT

Outcome Measures

Primary Outcome Measure:

1. clinical response rate
   Response is defined as a reduction of Pittsburgh sleep quality index (PSQI) global score by 3 points and more according to literature review

   [Time Frame: at 8 weeks from baseline]

Secondary Outcome Measure:

2. change of PSQI scores at the end of treatment
   PSQI scores will include PSQI global score, and scores of each domain (i.e. sleep duration, sleep disturbance, sleep latency, day dysfunction, sleep efficiency, overall sleep quality and sleep medication).

   [Time Frame: change from baseline PSQI scores at 8 weeks]

3. change of PSQI scores at the first followup
   PSQI scores will include PSQI global score, and scores of each domain (i.e. sleep duration, sleep disturbance, sleep latency, day dysfunction, sleep efficiency, overall sleep quality and sleep medication).

   [Time Frame: change from baseline PSQI scores at 12 weeks]

4. change of PSQI scores at the second followup
   PSQI scores will include PSQI global score, and scores of each domain (i.e. sleep duration, sleep disturbance, sleep latency, day dysfunction, sleep efficiency, overall sleep quality and sleep medication).

   [Time Frame: change from baseline PSQI scores at 16 weeks]

5. change of PSQI scores at the third followup
   PSQI scores will include PSQI global score, and scores of each domain (i.e. sleep duration, sleep disturbance, sleep latency, day dysfunction, sleep efficiency, overall sleep quality and sleep medication).

   [Time Frame: change from baseline PSQI scores at 20 weeks]
6. weekly dose of hypnotics
   If participants required hypnotic agents during the study because of unbearable sleep disorders, they will be allowed to take hypnotics initiating from the minimum dose and encouraged to complete the trial. The weekly dose of hypnotic agents will be recorded.

   [Time Frame: Day 0 (baseline), at 8 weeks (the end of treatment), at 12 weeks (the first followup),at 16 weeks (the second followup) and at 20 weeks (the third followup)]
7. adverse events
   Adverse events throughout the treatment and follow-up periods, regardless of its relevance to the interventions, will be documented and dealt with by appropriate measures.

   [Time Frame: through study completion, an average of 20 weeks]

Eligibility

Minimum Age: 18 Years

Maximum Age: 75 Years

Sex: All

Gender Based:

Accepts Healthy Volunteers: No

Criteria: Inclusion Criteria:

- Aged 18~75 years
- On regular dialysis (2 - 3 sessions weekly, 4 hours each session, total weekly dialysis hours ≥ 10 hours) for more than 3 months (but less than 10 years)
- Insomnia according to The Diagnostic and Statistical Manual of Mental Disorders, Fifth Edition (DSM-5)
- Global score of PSQI > 7
- Informed consent.

Exclusion Criteria:

- Presence of co-morbidities including cancer, congestive heart failure, connective tissue disease and hematologic diseases;
- Inadequately dialyzed, indicating by urea clearance index (KT/V) < 1.20;
- Presence of severe physical symptoms such as bone pain, itchy skin, sleep apnea and restless legs which are obviously causative for insomnia; and weary condition caused by severe anemia (hemoglobin<60g/L) or malnutrition (serum albumin<30g/L).
- Infections of external ears or malformed ears.

Contacts/Locations

Central Contact Person: Qizhan Lin, MD
Telephone: +86-020-81887233 Ext. 38502
Email: linqizhan656635@163.com

Central Contact Backup: Yuchi Wu, MD
Telephone: +86-020-81887233 Ext. 38501
Email: wuyuchi047@163.com

Study Officials: Qizhan Lin, MD
Study Principal Investigator
Guangdong Provincial Hospital of Traditional Chinese Medicine

Locations: China, Guangdong
Guangzhou HEMC (Higher Education Mega Center) Hospital
[Not yet recruiting]
Guangzhou, Guangdong, China, 510000
Contact: Daixin Zhao, PhD +86-020-39318101 greatdasin@163.com

Guangzhou Charity Hospital
[Not yet recruiting]
Guangzhou, Guangdong, China, 510370
Contact: Lixin Wang, PhD +86-13922797990 wanglixin1210@163.com
Contact: Hongyan Ma, MD +86-20-81499399 Ext. 8632 wtsh609@163.com

Guangzhou Hospital of Traditional Chinese Medicine
[Not yet recruiting]
Guangzhou, Guangdong, China, 510130
Contact: Xiangxin Meng, PhD +86-15989008896 mxx666@21cn.com

Wuyi Hospital of Traditional Chinese Medicine
[Not yet recruiting]
Jiangsu Sheng, Guangdong, China, 529099
Contact: Aicheng Yang, PhD +86-13500289361 easymu2008@163.com

Shenzhen Hospital of Traditional Chinese Medicine
[Not yet recruiting]
Shebu, Guangdong, China, 518026
Contact: Airong Qi, PhD +86-13715327780 81863418@163.com

Guangdong Provincial Hospital of Chinese Medicine
[Recruiting]
Guangzhou, Guangdong, China, 510120
Contact: Qizhan Lin, MD linqizhan656635@163.com

References

Citations:
[Study Results] Zou C, Yang L, Wu Y, Su G, Chen S, Guo X, Wu X, Liu X, Lin Q. Auricular acupressure on specific points for hemodialysis patients with insomnia: a pilot randomized controlled trial. PLoS One. 2015 Apr 15;10(4):e0122724. doi: 10.1371/journal.pone.0122724. eCollection 2015. PubMed 25874938

[Study Results] Wu Y, Zou C, Liu X, Wu X, Lin Q. Auricular acupressure helps improve sleep quality for severe insomnia in maintenance hemodialysis patients: a pilot study. J Altern Complement Med. 2014 May;20(5):356-63. doi: 10.1089/acm.2013.0319. Epub 2014 Feb 26. PubMed 24571603

Links: URL: http://journals.plos.org/plosone/article?id=10.1371/journal.pone.0122724
Description previously finished pilot RCT

URL: http://online.liebertpub.com/doi/abs/10.1089/acm.2013.0319
Description observational study on auricular acupressure

Study Data/Documents:
