# Supplementary material for: Auricular acupressure for insomnia in hemodialysis patients: study protocol for a randomized controlled trial
Source: Trials. 2018 Mar 7;19:171. doi: 10.1186/s13063-018-2546-2 (PMC5842538; doi:10.1186/s13063-018-2546-2)
Supplement: Supplementary file 4 — Informed consent materials. (DOC 30 kb) [file 13063_2018_2546_MOESM4_ESM.doc]

广东省中医院伦理委员会

Institutional Ethics Committee of Guangdong Provincial Hospital of Traditional Chinese Medicine

知情同意书·知情告知页（研究简介）

Information Leaflet for Informed Consent

亲爱的患者

我们了解到您因慢性肾功能衰竭行长期血液透析治疗。睡眠障碍是影响血液透析患者健康相关生活质量及长期存活率的一个重要影响因素。

我们将邀请您参加一项关于耳穴按压治疗血液透析患者睡眠障碍的疗效与安全性评价：一项多中心双盲随机对照试验.目的是验证耳穴按压干预血液透析患者失眠的临床疗效，为临床推广应用提供循证医学证据，并为进一步优化治疗方案提供思路。

在您决定是否参加这项研究之前，请尽可能仔细阅读以下内容，它可以帮助您了解该项研究以及为何要进行这项研究，研究的程序和期限，参加研究后可能给您带来的益处、风险和不适。如果您愿意，您可以请您的医生给予解释，或者可以和您的家属、朋友一起讨论，帮助您做出决定。

研究介绍

一、研究背景和研究目的

睡眠障碍是慢性肾脏病患者常见的并发症之一，尤其在血液透析患者中高发，其发生率可高达60-80%。睡眠障碍给慢性肾脏病患者的身心健康带来困扰，使人的生理节律紊乱，活动能力降低，严重影响患者生活质量。研究显示，睡眠障碍是影响血液透析患者健康相关生活质量及长期存活率的一个重要影响因素。

我们应用耳穴按压治疗帮助血液透析病人改善睡眠质量，初步观察到睡眠质量评分的改善和催眠药物的使用减少。然而，对于耳穴按压改善睡眠质量的确切作用及安全性，还需要进一步研究。

本研究将在3个血透中心进行，预计纳入合格受试者112名。

本项研究已经得到广东省中医院中医药科学技术研究专项批准并资助。广东省中医院伦理委员会已经审议此项研究是遵从赫尔辛基宣言原则，符合医学伦理的。

二、哪些人不宜参加研究

①合并严重的未控制的心、脑、肝或造血系统等其它系统原发性疾病或恶性肿瘤者；

②透析不充分，Kt/V ＜1.20；

③存在明显的躯体症状如疼痛、瘙痒、睡眠呼吸暂停、不宁腿综合征等可以解释失眠的临床情况，以及严重贫血（血红蛋白< 60g/L）或营养不良（血清白蛋白< 30g/L）导致的疲乏。

三、如果参加研究将需要做什么

1、在您入选研究前，您将接受以下检查以确定您是否可以参加研究

医生将询问、记录您的病史，对您进行全面的体格检查。

您需要进行血常规，肝功能，肾功能等理化检查及透析充分性评估。

若您以上检查合格，将按以下步骤进行研究

①研究员将通过问卷调查或访谈的方式了解您的睡眠质量和生活质量。

②您将被随机分配进入某个组，从而接受不同的治疗方案。您和您接诊医生均无法事先知道会被分配进哪个组。

③您需要独立完成一份睡眠日志。

3.需要您配合的其他事项

您的随访非常重要。您需要按医生和您的约定的时间来医院就诊。

在研究期间您无需刻意改变您的生活方式或治疗方案，但我们建议您在主管医师的指导下规范合理用药，并尽量完整地记录（或向医师汇报）您的用药情况。同时避免烟、酒、咖啡等刺激性食物。

四、参加研究可能的受益

您和社会将可能从本项研究中受益。此种受益包括您的病情得到关注，通过生活方式调整或药物治疗有可能获得改善，以及本项研究可能有一些新的发现，为以后提出新的治疗方案，使得其他与您存在相似病情的其他病人同样获得益处。

五、参加研究可能的不良反应、风险和不适、不方便

本研究为随机对照临床试验，涉及的干预措施为非药物疗法——耳穴按压。我们已通过临床实践和初期的临床观察研究发现该疗法是安全的，且对睡眠质量改善具有潜在价值。

如果在研究中您出现任何不适，或病情发生新的变化，或任何意外情况，不管是否与治疗方法有关，均应及时通知您的医生，他/她将对此作出判断和医疗处理。

您在研究期间需要按时到医院随访，做一些理化检查，或在您透析治疗期间对您进行病情方面的询问，这些都有可能给您造成麻烦或带来不便。若您确实不能配合，您可以告知研究者，我们会根据您的具体情况进行调整。

六、有关费用

研究者将支付您参加本项研究期间所做的与研究有关治疗(耳穴按压治疗)的费用，本项研究不额外增加您的医疗费用。

如果您同时合并其他疾病所需的治疗和检查，将不在免费的范围之内。

七、个人信息保密的吗？

您的医疗记录（研究病历/CRF、化验单等）将完整地保存在医院，医生会将化验检查结果记录在您的门诊病历上。研究者、申办者代表和伦理委员会将被允许查阅您的医疗记录。任何有关本项研究结果的公开报告将不会批露您的个人身份。我们将在法律允许的范围内，尽一切努力保护您个人医疗资料的隐私。除本研究外，有可能在今后的其他研究中会再次利用您的医疗记录和病理检查标本。

八、怎样获得更多的信息？

您可以在任何时间提出有关本项研究的任何问题。您的医生或研究者将给您留下他/她的电话号码以便能回答您的问题。

如果您对参加研究有任何抱怨，请联系伦理委员会办公室。

如果在研究过程中有任何重要的新信息，可能影响您继续参加研究的意愿时，您的医生会及时通知您。

九、可以自愿选择参加研究和中途退出研究

是否参加研究完全取决于您的自愿。您可以拒绝参加此项研究，或在研究过程中的任何时间退出本研究，这都不会影响您和医生间的关系，都不会影响对您的医疗有其他方面利益的损失。

您的医生或研究者出于对您的最大利益考虑，可能会随时终止您参加本项研究。

如果您不参加本项研究，或中途退出研究，并不会影响医师对您的诊疗。您不必因有此顾虑而选择参加本项研究。

如果您因为任何原因从研究中退出，您可能被询问有关您使用试验方法的情况。如果医生认为需要，您可能被要求进行实验室检查和体格检查。这对保护您的健康十分有利。

十、现在该做什么？

在您做出参加研究的决定前，请尽可能向您的医生询问有关问题，直至您对本项研究完全理解。

是否参加本项研究由您自己决定。您可以和您的家人或者朋友讨论后再做出决定。

感谢您阅读以上材料。如果您决定参加本项研究，请告诉您的医生或研究助理，他她会为您安排一切有关研究的事务。

请您保留这份资料。

知情同意书·同意签字页

Signature Leaflet for Informed Consent

**临床研究项目名称**：耳穴按压治疗血液透析患者睡眠障碍的疗效与安全性评价：一项多中心双盲随机对照试验

**申办者**：广东省中医院

**有关课题资助单位的任务下达文件证明**：广东省中医院中医药科学技术研究专项（No.YN2015MS25）

**伦理审查批件号**：

同意声明

我已经阅读了上述有关本研究的介绍，而且有机会就此项研究与医生讨论并提出问题。我提出的所有问题都得到了满意的答复。

我知道参加本研究可能产生的风险和受益。我知晓参加研究是自愿的，我确认已有充足时间对此进行考虑，而且明白：

● 我随时可以向医生咨询更多的信息。

● 我可以随时退出本研究，而且不会受到歧视或报复，医疗待遇与权益不会受到影响。

我同样清楚，如果我中途退出本研究，特别是由于药物的原因使我退出研究时，我若将病情变化告诉医生，完成相应的体格检查和理化检查，这将对我本人和整个研究十分有利。

如果因患病我需要采取任何其他的药物治疗，我会在事先征求医生的意见，或在事后如实告诉医生。

我同意药品监督管理部门、伦理委员会或申办者代表查阅我的研究资料。

我同意□ 或拒绝□ 除本研究以外的其他研究利用我的医疗记录和病理检查标本。

我将获得一份经过签名并注明日期的知情同意书副本。

最后，我决定同意参加本项研究。

患者/受试者 签名： ––––年––月––日

监护人/授权委托人 签名： ––––年––月––日

联系电话： 手机号：

我确认已向患者解释了本试验的详细情况，包括其权利以及可能的受益和风险，并给其一份签署过的知情同意书副本。

医生签名： 日期： 年 月 日

医生的工作电话： 手机号：

研究者办公室联系电话：020-81887233-38502

广东省中医院伦理委员会办公室联系电话：020-81887233-35943
